# Immunohistochemical determination of the miR-1290 target *arylamine N-acetyltransferase 1 (NAT1)* as a prognostic biomarker in breast cancer

**DOI:** 10.1186/1471-2407-14-990

**Published:** 2014-12-20

**Authors:** Yumi Endo, Hiroko Yamashita, Satoru Takahashi, Shinya Sato, Nobuyasu Yoshimoto, Tomoko Asano, Yukari Hato, Yu Dong, Yoshitaka Fujii, Tatsuya Toyama

**Affiliations:** Department of Oncology, Immunology and Surgery, Nagoya City University Graduate School of Medical Sciences, 1 Kawasumi, Mizuho-cho, Mizuho-ku, Nagoya, 467-8601 Japan; Department of Experimental Pathology and Tumor Biology, Nagoya City University Graduate School of Medical Sciences, Kawasumi, Mizuho-cho, Mizuho-ku, Nagoya, 467-8601 Japan; Department of Breast and Endocrine Surgery, Hokkaido University Hospital, Kita 14, Nishi 5, Sapporo, 060-8648 Japan

**Keywords:** Breast cancer, miR-1290, Arylamine N-acetyltransferase 1 (NAT1)

## Abstract

**Background:**

There are many molecular differences between estrogen receptor α (ERα)-positive and ER-negative breast cancers. Recent analyses have shown that the former can be divided into two subtypes, luminal A and luminal B. These differ in response to endocrine therapy and chemotherapy, and in prognosis. In a previous study, we found that microRNA (miR)-1290 that was significantly down-regulated in luminal A tumors and its potential target *arylamine N-acetyltransferase 1 (NAT1)*. The aim of the present study was to determine whether *NAT1* is a *bona fide* target of miR-1290, and to investigate the impact of NAT1 on breast cancer prognosis.

**Methods:**

Luciferase reporter assays were employed to validate *NAT1* as a putative miR-1290 target gene. Expression of NAT1, ERα, progesterone receptor (PgR) and HER2 was analyzed in 394 breast cancer samples by immunohistochemistry.

**Results:**

*NAT1* was confirmed to be a direct target of miR-1290. Levels of expression of NAT1 were positively correlated with those of ERα (*P* < 0.0001) and PgR (*P* < 0.0001), but negatively correlated with both tumor grade and size (*P* < 0.0001). Kaplan-Meier analysis showed that the presence of NAT1 was significantly associated with increased overall survival (OS) (*P* = 0.0416) in these patients. Similarly, significant associations of NAT1 with disease-free survival (DFS) (*P* = 0.0048) and OS (*P* = 0.0055) in those patients who received adjuvant endocrine therapy with tamoxifen (n = 176) were found. Moreover, NAT1 was also significantly associated with increased DFS (*P* = 0.0025) and OS (*P* = 0.0007) in the subset of lymph node-positive patients (n = 147). Univariate and multivariate analyses showed significant associations between levels of NAT1 and DFS (*P* = 0.0005 and 0.019, respectively).

**Conclusions:**

We report that miR-1290 directly targets the NAT1 3′-UTR and that NAT1 protein expression is correlated with improved OS of breast cancer patients. NAT1 is a possible prognostic biomarker for lymph node-positive breast cancer. Thus, miR-1290 and its target NAT1 are associated with important characteristics of breast cancer.

**Electronic supplementary material:**

The online version of this article (doi:10.1186/1471-2407-14-990) contains supplementary material, which is available to authorized users.

## Background

Breast cancers represent a heterogeneous group of tumors that are diverse in behavior, outcome, and sensitivity to therapy. Emerging data demonstrate that stratification of tumors by gene expression profiles can divide breast cancer into five main subtypes which are associated with different clinical outcomes. Two of these are estrogen receptor (ER)-positive (luminal A and luminal B) and three are ER-negative (basal-like, HER2 positive, normal breast-like) [1, 2].

Blenkiron and colleagues analyzed microRNA (miRNA) expression in human breast cancer, and reported that many miRNAs were differentially expressed between breast cancer subtypes including luminal A and luminal B. They also reported an association between miRNA expression profiling and clinicopathological factors such as ERα status and tumor grade [3]. miRNAs are a class of naturally occurring small non-coding RNAs that control gene expression by targeting mRNAs for translational repression or cleavage [4]. Mature miRNAs recognize sites in the 3′-untranslated regions (UTR) of the target mRNAs and cause mRNA degradation or translational repression. miRNAs have been characterized as oncogenic, tumor suppressors or as components of regulatory pathways critical for tumorigenesis [4, 5].

In our previous study, we reported a miRNA, miR-1290, potentially differentiating between luminal A and luminal B/HER2-negative tumors. We compared expression profiles of miRNAs and mRNAs from ER^high^ Ki67^low^ and ER^low^ Ki67^high^ tumors, which are considered typically luminal A and luminal B/HER2-negative, respectively. We also found 4 potential target genes (*FOXA1*, *arylamine N-acetyltransferase 1 (NAT1)*, *BCL2* and *MAPT*) of miR-1290 [6]. Transfection experiments revealed that transfection of ER-positive breast cancer cells with miRNA-1290 resulted in decreased expression of *NAT1* and *FOXA1* mRNA but not the other two potential target genes. Moreover, Western blot analysis showed that miR-1290 induced a dose-dependent decrease in NAT1 protein expression. Of these potential target genes, *NAT1* is the most promising target of miR-1290 [6].

Arylamine N-acetyltransferases (NATs) are present in many species. NATs are cytosolic conjugating enzymes which transfer an acetyl group from acetylCoenzyme A to a xenobiotic acceptor substrate. Human NATs were originally identified as drug-metabolizing enzymes [7–9]. Recent studies focused on their role in the activation and detoxification of environmental carcinogens and implicated human NATs in cancer and in development [7, 8, 10, 11]. The human NAT gene products NAT1 and NAT2 have distinct substrate specificities: NAT2 acetylates hydralazine and NAT1 acetyates p-aminosalicylate (p-AS) and the folate catabolite p-aminobenzoylglutamate (p-abaglu). Human NAT2 is mainly present in liver and gut, whereas human NAT1 and its murine homologue are present in many adult tissues and in early embryos [12]. *NAT1* is one of the most highly overexpressed genes in ER-positive relative to ER-negative breast tumors [1, 12, 13]. Moreover, *NAT1* is one of a cluster of genes including the highly expressed ER in luminal A tumors [2].

The aim of the present study was to clarify whether *NAT1* is a *bona fide* target of miR-1290 and to investigate the impact of NAT1 expression on breast cancer prognosis.

## Methods

### Cell culture and transfections

COS-7 cells (American Type Culture Collection; ATCC) were grown in RPMI 1640 containing 10% fetal bovine serum (FBS), 2 mmol/L L-glutamine and penicillin-streptomycin (50 IU/mL and 50 mg/mL, respectively), at 37°C with 5% CO2. Transfections of pre-miR-1290 precursor (hsa-miR-1290; Ambion Inc., Austin, USA) were performed with Cell Line Nucleofector kits (Amaxa Biosystems, Cologne, Germany) using a Nucleofector device (Amaxa Biosystems) according to the manufacturer’s instructions [14]. A nonspecific control miRNA (Pre-miR miRNA Negative Control #2; Ambion Inc.) was used as a negative control.

### Dual-luciferase reporter assay

The region of human *NAT1*-3′UTR (bases 52478 to 53073) containing two putative miR-1290-binding sites, was amplified from MCF7 cells using the PCR primers listed in Additional file 1: Table S1, and cloned into the pMIR-report™ luciferase plasmid (Ambion, Austin TX); these were designated NAT1-wt. Three derivative constructs of NAT1-wt with mutations in the putative miR-1290-binding sites were generated using a QuikChange II XL Site-Directed Mutagenesis Kit (Agilent Technologies) and the primers listed in Additional file 1: Table S1, and were designated NAT1-mut1, -mut2, and -mut1 + 2. All of the constructs were verified by direct sequencing. Pre-miR-1290 precursor and a nonspecific control miRNA were co-transfected with 3 μg each of the reporter vector constructs and an internal control vector (pGL4.74, Promega) into COS-7 cells (1 × 10^6^ cells) in a 24-well format. Luciferase activity was measured 24 hours later using a dual-luciferase reporter assay system (Promega) and a Lumat LB9507 luminometer (Berthold Technologies, Germany). The firefly luciferase activities of the reporter constructs were normalized against the renilla luciferase activities of the internal control vector. The degree of reduction of luciferase activity relative to the samples transfected with nonspecific control miRNA was taken as an index of the effect of the miR-1290 on the post-transcriptional regulation of the *NAT1* gene.

### Patients and breast cancer tissue

Breast tumor specimens from 394 female patients with invasive breast carcinoma who were treated at Nagoya City University Hospital between 1995 and 2009 were included in the study (Table 1). This protocol was approved by the Institutional Review Board of Nagoya City University Graduate School of Medical Sciences and conformed to the guidelines of the 1996 Declaration of Helsinki. Written informed consent for the use of the surgically-resected tumor tissues was provided by all patients prior to treatment. The samples were chosen from a continuous series of invasive carcinomas. All patients underwent surgical treatment (mastectomy or lumpectomy). Patients received appropriate adjuvant endocrine or chemotherapy for metastatic disease (Table 1).

**Table 1:** **Clinicopathological characteristics of patients**

	Total
No. of patients	394
Age (years)	
Mean ± SD	57.0 ± 12.8
Range	28-94
Tumor size (cm)	
≤2.0	197 (50.0%)
2.1-5.0	183 (46.5%)
>5.0	12 (3.1%)
Unknown	2 (0.4%)
No. of positive lymph nodes	
0	207 (52.5%)
1-3	108 (27.4%)
4-9	23 (5.9%)
≥10	15 (3.8%)
Unknown	41 (10.4%)
Tumor grade	
1	111 (28.2%)
2	113 (28.7%)
3	146 (37.1%)
Unknown	24 (6.0%)
ERα	
Negative	31(7.9%)
Positive	363 (92.1%)
PgR	
Negative	86 (21.8%)
Positive	306 (77.7%)
Unknown	2 (0.5%)
HER2 status	
Negative	278 (70.6%)
Positive	41 (10.4%)
Unknown	75 (19.0%)
Adjuvant therapy	
None	30 (7.6%)
Endocrine therapy	177 (44.9%)
Chemotherapy	29 (7.4%)
Combined	155 (39.3%)
Unknown	3 (0.8%)

### Immunohistochemistry (IHC)

Tissue microarrays were constructed using paraffin-embedded, formalin-fixed tissue from 394 breast cancer samples. Tissue array sections were immunostained with 4 commercially available antibodies using the Bond-Max Autostainer (Leica Microsystems, Newcastle, UK) and the associated Bond Refine Polymer Detection kit [15]. Primary antibodies included mouse monoclonal anti-human ERα antibody (1D5, Dako, Glostrup, Denmark) at 1:100 dilution, mouse monoclonal anti-human PgR antibody (636, Dako) at 1:100 dilution and rabbit polyclonal anti-human NAT1 antibody (ab92785, Abcam) at 1:100 dilution. The expression of ERα and PgR was scored by assigning proportion and intensity scores, according to Allred’s procedure [16]. In brief, a proportion score represented the number of tumor cells staining positive as follows: 0 (none), 1 (<1/100), 2 (1/100 to 1/10), 3 (1/10 to 1/3), 4 (1/3 to 2/3), and 5 (>2/3). Any brown nuclear staining in the breast epithelium was counted towards the proportion score. An intensity score represented the average intensity of the positive cells as follows: 0 (none), 1 (weak), 2 (intermediate), 3 (strong). The proportion and intensity scores were then added to obtain a total score ranging from 0 to 8. Staining status by IHC was then assessed as negative (scores 0, 2) or positive (scores 3–8) [17]. Immunostaining of HER2 was evaluated using the HercepTest (Dako). To determine the level of HER2 expression, the membrane staining pattern was estimated and scored on a scale of 0 to 3+. Tumors with a score of 2+ were tested for gene amplification by fluorescence in situ hybridization (FISH) using the PathVysion assay (Vysis, Abbott Laboratories, Abbott Park, IL). A ratio ≥2.0 for HER2 gene/chromosome 17 was considered positive. Tumors were considered HER2-positive if IHC staining was 3+ or they were FISH-positive [18]. NAT1 expression level was assessed as the percentage of stained tumor cells (Additional file 2: Figure S1). Tumors with at least one NAT1**-**positive tumor cell were considered to indicate the presence of this protein. The cutoff points for the expression levels of NAT1 were set at least one stained cell, which allowed us to obtain the most significant difference between patient groups in prognostic analyses.

### Statistical analysis

Results are expressed as the mean ± S.E. Student’s t test was used to compare data between two groups. *P* values less than 0.05 were considered to be statistically significant. Estimation of disease-free survival and overall survival was performed using the Kaplan-Meier method, and differences between survival curves were assessed with the Wilcoxon test. Cox’s proportional hazards model was used for univariate and multivariate analyses of prognostic values. JMP SAS software (SAS Institute Japan) was used for data analysis.

## Results

### Mir-1290 targets the *NAT1*3′-UTR directly

**Figure 1:**
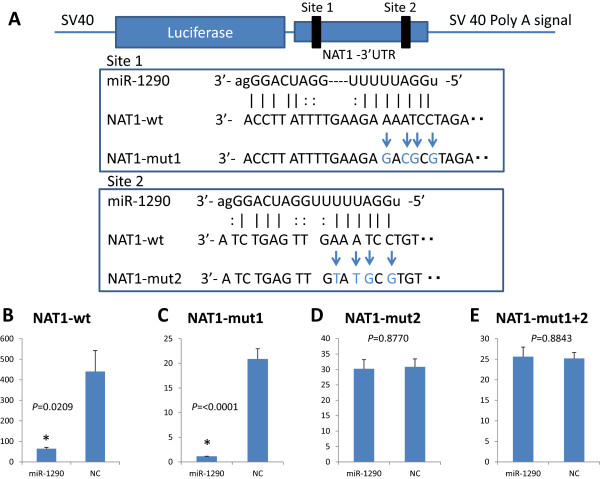
**Identification of miR-1290 target sites in the NAT1 3′-UTR. A**, Schematic of the 3′-UTR-containing reporter constructs for potential miR-1290 target sites in NAT1. The 3′-UTR of the NAT1 gene was inserted just downstream of the firefly luciferase gene in the pMIR-report luciferase plasmid (NAT1-wt). Next, the mutated derivatives (Nat1-mut1, -mut2 and –mut1 + 2) of NAT1-wt were generated by inserting mutations into two putative binding sites corresponding to the seed-sequence of miR-1290. **B-E**, Cells were transfected with either miR-1290 or nonspecific control miRNA (NC). Luciferase activity was assayed 24 hr later. The data are shown as luciferase activity relative to the vehicle (pGL4.74).

Two sites in the *NAT1* 3′-UTR were predicted to be potential target sites of miR-1290 according to miRanda (http://www.microrna.org/). To determine whether *NAT1* is a direct target of miR-1290, we cloned its 3′-UTR into a pMIR-report™ luciferase plasmid to perform a reporter assay (Figure 1A). When miR-1290 precursor was transfected into the cells together with this reporter construct, luciferase activity was repressed relative to the nonspecific control miRNA (Figure 1B). Furthermore, we cloned each putative miR-1290-target site having multiple mutants in their sequences (Figure 1A) that corresponded to the “seed sequence” of miR-1290 into the pMIR-report plasmid and performed reporter assays (Figure 1C-E). When miR-1290 precursor was transfected into cells with the NAT1-mut1, luciferase activity was still repressed (Figure 1C), whereas this was no longer the case for NAT1-mut2 or –mut1 + 2 (Figure 1D, E). These results suggest that site 2 in the *NAT1* 3′-UTR is the putative target site of miR-1290.

### Expression of NAT1 protein and its relationship with clinicopathological factors

The expression levels of NAT1 protein in breast cancer tissues was examined by immunohistochemistry (IHC). Levels of NAT1 were positively correlated with ERα (*P* < 0.0001) and PgR (*P* < 0.0001), but negatively correlated with tumor grade and size (*P* < 0.0001) (Table 2).

**Table 2:** **Correlation between expression levels of NAT1 and clinicopathological fators (n = 394)**

	ER	PgR	HER2 status	Tumor grade	Tumor size	No. of positive lymph nodes
	(Allred score)	(Allred score)				
NAT1	^a^+ 0.286	+ 0.356	- 0.095	- 0.405**	- 0.247	- 0.031
	^b^< 0.0001*	<0.0001*	0.113	<0.0001*	<0.0001*	0.573

^a^Speaman’s correlation coefficient.

^b^ *P*, speaman’s rank correlation test.

*P < 0.05 is considered significant.

**spearman’s correlation coefficient greater than +0.40 or less than -0.40 have strongly correlate.

### The presence of NAT1 is correlated with improved overall survival

**Figure 2:**
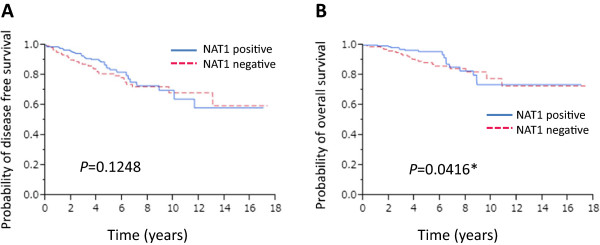
**Kaplan-Meier survival analyses of the 394 breast cancer patients.** Disease-free survival **(A)** and overall survival **(B)** of the 394 breast cancer patients stratified according to the presence or absence of NAT1 protein.

**Figure 3:**
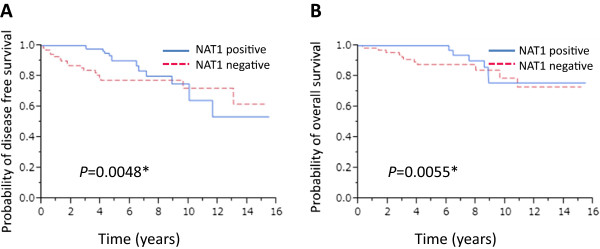
**Kaplan-Meier survival analyses of the patients who received adjuvant endocrine therapy with Tamoxifen.** Disease-free survival **(A)** and overall survival **(B)** of the 176 patients who received adjuvant endocrine therapy with tamoxifen stratified according to expression of NAT1.

We next analyzed the correlation between the presence of NAT1 protein in breast cancer tissues and patient prognosis. Kaplan-Meier analysis of all 394 patients together showed that the presence of NAT1 was not strongly associated with disease-free or overall survival (OS), although the latter did show marginal significance (P = 0.0416) (Figure 2B). We then investigated the correlation between the presence of NAT1 and prognosis in ERα-positive patients (n = 363). We found no association between the presence of NAT1 and favorable disease-free survival (DFS) (*P* = 0.3461) and OS (*P* = 0.1319) (data not shown). However, Kaplan-Meier analysis showed that the presence of NAT1 was significantly associated with favorable DFS (*P* = 0.0048) and OS (*P* = 0.0055) in patients who received adjuvant endocrine therapy with tamoxifen (n = 176) (Figure 3A and B).

### NAT1 protein expression is prognostic for lymph node-positive breast cancer

**Figure 4:**
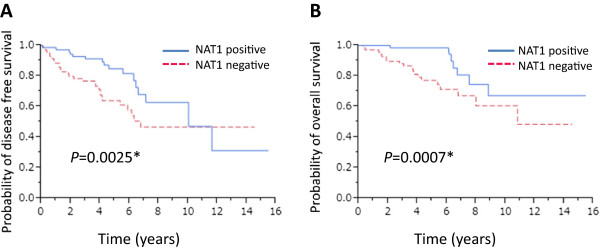
**Kaplan-Meier survival analyses of the lymph node-positive breast cancer patients.** Disease-free survival **(A)** and overall survival **(B)** of the 147 lymph node-positive breast cancer patients stratified according to expression of NAT1.

We then analyzed NAT1 in tumors from lymph node-negative (n = 247) and lymph node-positive patients (n = 147). Kaplan-Meier analysis showed that the presence of NAT1 was significantly associated with favorable DFS (*P* = 0.0025) and OS (*P* = 0.0007) in lymph node-positive (Figure 4A and B), but not -negative patients (Additional file 3: Figure S2). Univariate analysis revealed significant associations between levels of expression of ERα (*P* = 0.0393), the levels of expression of NAT1 (*P* = 0.0005), tumor size (*P* = 0.0028), number of positive lymph nodes (*P* = 0.0006) and DFS. Furthermore, NAT1 (*P* = 0.019) and the number of positive lymph nodes (*P* = 0.0122) remained significant when assessed by multivariate analysis (Table 3). Univariate analysis indicated significant associations between levels of ERα (*P* = 0.0034), PgR (*P* = 0.0221), NAT1 (*P* = 0.0054), tumor size (*P* = 0.0188), number of positive lymph nodes (*P* = 0.0048) and OS. In multivariate analysis, only NAT1 tended to associate with favorable OS but this was not significant (*P* = 0.0925) (Table 4).

**Table 3:** **Univariate and multivariate analysis of factors predicting disease free survival (LN+, n = 147)**

	Univariate			Multivariate		
	RR	95% CI	P	RR	95% CI	P
ER (Allred score)	0.881	0.789-0.949	0.0393*	0.915	0.817-1.034	0.1491
PgR (Allred score)	0.927	0.839-1.027	0.1456			
HER2	1.174	0.684-1.849	0.5326			
NAT1 (%)	0.967	0.940-0.987	0.0005*	0.974	0.946-0.996	0.019*
Tumor grade	1.24	0.810-1.936	0.3255			
Tumor size	1.367	1.120-1.645	0.0028*	1.048	0.822-1.319	0.698
No. of positive lymph nodes	1.092	1.042-1.137	0.0006*	1.076	1.017-1.131	0.0122*

Cl, cofidence interval. ^*^ *P* < 0.05.

**Table 4:** **Univariate and multivariate analysis of factors predicting overall survival (LN+, n = 147)**

	Univariate			Multivariate		
	RR	95% CI	P	RR	95% CI	P
ER (Allred score)	0.804	0.704-0.927	0.0034*	0.834	0.704-0.985	0.0324*
PgR (Allred score)	0.858	0.753-0.978	0.0221*	0.989	0.845-1.165	0.8875
HER2	1.21	0.569-2.234	0.5865			
NAT1 (%)	0.961	0.914-0.990	0.0054*	0.973	0.925-1.005	0.0925
Tumor grade	1.655	0.911-3.211	0.0998			
Tumor size	1.372	1.058-1.729	0.0188*	1.045	0.772-1.381	0.7656
No. of positive lymph nodes	1.097	1.032-1.156	0.0048*	1.09	1.014-1.165	0.0208*

Cl, cofidence interval. ^*^ *P* < 0.05.

## Discussion

We focused on microRNAs and on two different subtypes of ER-positive breast cancer, and found that miR-1290 and its potential target *NAT1* may be informative for patient survival. We demonstrated that miR-1290 directly down-regulates NAT1 expression. In addition, we found that breast cancer patients with tumors expressing NAT1 tended to have better overall survival than those whose tumors were NAT1-negative. Furthermore, in lymph node-positive patients, the presence of NAT1 was significantly associated with favorable DFS and OS.

Although there are two predicted miR-1290 target sites in the *NAT1* 3′-UTR, we found that only site 2 was the likely target site. The role of miR-1290 has not yet been analyzed, but it was reported as one of the differentially expressed miRNAs in various cancers, although not in breast cancer [19, 20]. Wu and colleagues reported that miR-1290 was significantly up-regulated in colon cancer tissues and that its up-regulation postponed cytokinesis and led to the formation of multinucleated cells. Moreover, they reported that the enforced expression of miR-1290 activated the Wnt pathway and increased the levels of reprogramming-related transcription factors c-Myc and Nanog [21]. Recently, Li and colleagues reported that serum miR-1290 levels distinguished patients with low-stage pancreatic cancer from healthy controls [22]. In a previous study, miR-1290 expression was strongly down-regulated in luminal A tumors and was positively correlated with tumor grade.

NATs are polymorphic drug-metabolizing enzymes [12]. There are two closely related genes on chromosome 8 that encode the two human NATs – NAT1 and NAT2 [7]. Human NAT1 and its murine homologue are present in many adult tissues including breast tissue, as well as in early embryos. Human NAT1 acetylates p-AS and the folate catabolite p-ABG [8, 12], and may contribute to folate and acetylCoA homeostasis. NAT1 is represented on most microarray chips, so interrogation of public databases has revealed changes in NAT1 mRNA levels associated with different cancers and cancer subtypes [7]. Regarding breast cancer, several independent studies showed that NAT1 expression clustered with expression of the estrogen receptor [1, 2]. The positive association of NAT1 and estrogen receptor was strengthened by Adam et al. who showed by immunohistochemistry that NAT1 protein levels were higher in estrogen receptor-positive than negative breast cancer tissue [23]. In agreement with these data, in the present study, we showed that expression of NAT1 was positively correlated with ERα (*P* < 0.0001).

We showed that NAT1 protein expression was a prognostic marker in breast cancer patients, which supports previous reports by other groups. High expression of NAT1 has been shown to correlate with better outcome among ER-positive breast cancers [24, 25]. Furthermore, in a study of primary male breast cancers, NAT1 positivity was reported to be correlated with better outcome [26]. Bieche and colleagues reported a positive correlation between *NAT1* mRNA levels and ERα mRNA levels [24]. One of the reasons for the association between NAT1 and good prognosis might be the role of NAT1 as a drug metabolizing enzyme. Bieche and colleagues reported high NAT1 could predict the response to tamoxifen in ER-positive breast cancer [24] and hypothesized that strong intratumoral NAT1 expression could lead to increased detoxification of genotoxic and/or estrogenic tamoxifen metabolites. In addition, Kim and colleagues reported that the NAT1 methylation rate was lower in a control group than in a tamoxifen-resistant group, and that the expression of *NAT1* mRNA was lower in the latter [27]. Therefore, it seems that NAT1 has an important role in the response to tamoxifen. In the present study, we also showed that NAT1 was significantly associated with favorable DFS and OS in patients who received adjuvant endocrine therapy with tamoxifen. Moreover, we showed that patients whose tumors were NAT1-positive had a significantly more favorable prognosis in node-positive breast cancer patients. Almost all patients (97.3%, 143/147) with metastatic invaded lymph nodes received adjuvant systemic therapy, not only with tamoxifen but also other hormonal- and chemo-therapies. Although patients with positive lymph nodes are at high risk of recurrence, the effect of the adjuvant systemic therapy is considered to be more important in node-positive than in node-negative patients. NAT1 might influence not only the metabolism of tamoxifen but also other drugs. We also showed that expression of NAT1 correlated positively with expression of ERα, which might be another reason for the correlation between the presence of NAT1 and good prognosis.

In contrast, NAT1 overexpression can lead to resistance to certain drugs. Using nontransformed breast epithelial HB4a cells, Adam and colleagues reported that NAT1 overexpression conferred a growth and survival advantage, even in low serum concentrations [23]. Moreover, these cells were more resistant to etoposide-induced cell death, prompting the authors to suggest that NAT1 may have indirect oncogenic effects. Similarly, NAT1 expression was higher in gemcitabine-resistant Calu3 cells than in sensitive cells. Although we used different drugs, our results are in contrast to some previous hypotheses. This is a limitation of this study.

In addition, there have been some reports that NAT1 has an important role in cancer cell biology. The small molecule inhibitor Rhod-o-hp was used to investigate the effect of NAT1 inhibition in MDA-MB-231 breast cancer cells. This resulted in changes in cell proliferation rates and invasiveness [28]. Moreover, knockdown of NAT1 expression using short-hairpin RNA (shRNA) in the noninvasive HT-29 colon cancer cell line resulted in a marked change in cell morphology that was accompanied by an increase in cell-cell contact inhibition of growth and a loss of cell viability at confluence [29]. These reports pointed to NAT1 as a novel target for anticancer drug development. NAT1 might play a role as a predictive factor for therapeutic effects and act as a therapeutic target, similar to the ER.

## Conclusions

This study demonstrated that miR-1290 directly targets the *NAT1* 3′-UTR. We showed that levels of expression of NAT1 were positively correlated with ERα and PgR, but negatively correlated with tumor grade and size. Kaplan-Meier analysis showed that the presence of NAT1 was significantly associated with increased OS in breast cancer patients and with DFS and OS in patients who received adjuvant endocrine therapy with tamoxifen. Moreover, NAT1 was more significantly associated with increased DFS and OS in lymph node-positive breast cancer patients. Univariate and multivariate analyses showed significant associations between levels of NAT1 and DFS. We conclude that NAT1 might be a suitable DFS prognostic biomarker, particularly for lymph node-positive breast cancer. Thus, miR-1290 and its potential target NAT1 are associated with characteristics of breast cancer.

## Electronic supplementary material

Additional file 1: Table S1: Primer sequences for generating luciferase reporter constructs. (DOC 30 KB)

Additional file 2: Figure S1: NAT1 immunohistochemical staining of the breast cancer tissues. A, NAT1 expression level was assessed as 0 percent of positively stained tumor cells. B, NAT1 expression level was assessed as 12%. C, NAT1 expression level was assessed as 50%. D, NAT1 expression level was assessed as 75%. x400. (PPTX 6 MB)

Additional file 3: Figure S2: Kaplan-Meier survival analyses of the lymph node negative breast cancer patients. Disease free survival (A) and overall survival (B) of the 161 lymph node positive breast cancer patients stratified according to the presence or absence of NAT1 protein. (PPTX 71 KB)

## Abbreviations

DFS: Disease-free survival
EIA: Enzyme immunoassay
ERα: Estrogen receptor α
FISH: Fluorescence in situ hybridization
IHC: Immunohistochemistry
NAT1: Arylamine N-acetyltransferase 1
NATs: Arylamine N-acetyltransferases
miRNA: microRNA
OS: Overall survival
PgR: Progesterone receptor
UTR: Untranslated regions.
