# Supplementary material for: Immunohistochemical determination of the miR-1290 target arylamine N-acetyltransferase 1 (NAT1) as a prognostic biomarker in breast cancer
Source: BMC Cancer. 2014 Dec 20;14:990. doi: 10.1186/1471-2407-14-990 (PMC4364092; doi:10.1186/1471-2407-14-990)
Supplement: Supplementary file 1 — Additional file 1: Table S1: Primer sequences for generating luciferase reporter constructs. (DOC 30 KB) [file 12885_2014_5180_MOESM1_ESM.doc]

Additiona file 1: Table S1. Primer sequences for generating luciferase reporter constructs.

| Primer name | Sequence (5'->3') | Purpouse |
| --- | --- | --- |
| Spe1-hNAT1-F | CTTGTCTACTAGT* CATCCAGCTCACCAG | cloning |
| Sac1-hNAT1-R | TAGGGAGTCAGAGCTC* CAGATCTAAAAA | cloning |
| hNAT1-Target1-F | CCTTACCTTATTTTGAAGAGACGCG**TAGACATCAAATCATTTCAC | mutagenesis |
| hNAT1-Target1-R | GTGAAATGATTTGATGTCTACGCGTC**TCTTCAAAATAAGGTAAGG | mutagenesis |
| hNAT1-Target2-F | GTGTAGATCTGAGTTGTATGCG**TGTGGACACTGGGCGAATTAC | mutagenesis |
| hNAT1-Target2-R | GTAATTCGCCCAGTGTCCACACGCATA**CAACTCAGATCTACAC | mutagenesis |

Under line * binding site of restriction enzymes

Under line **mutagenesis site
