# Supplementary figures and images for: Immunohistochemical determination of the miR-1290 target arylamine N-acetyltransferase 1 (NAT1) as a prognostic biomarker in breast cancer
Source: BMC Cancer. 2014 Dec 20;14:990. doi: 10.1186/1471-2407-14-990 (PMC4364092; doi:10.1186/1471-2407-14-990)

## Slide 1
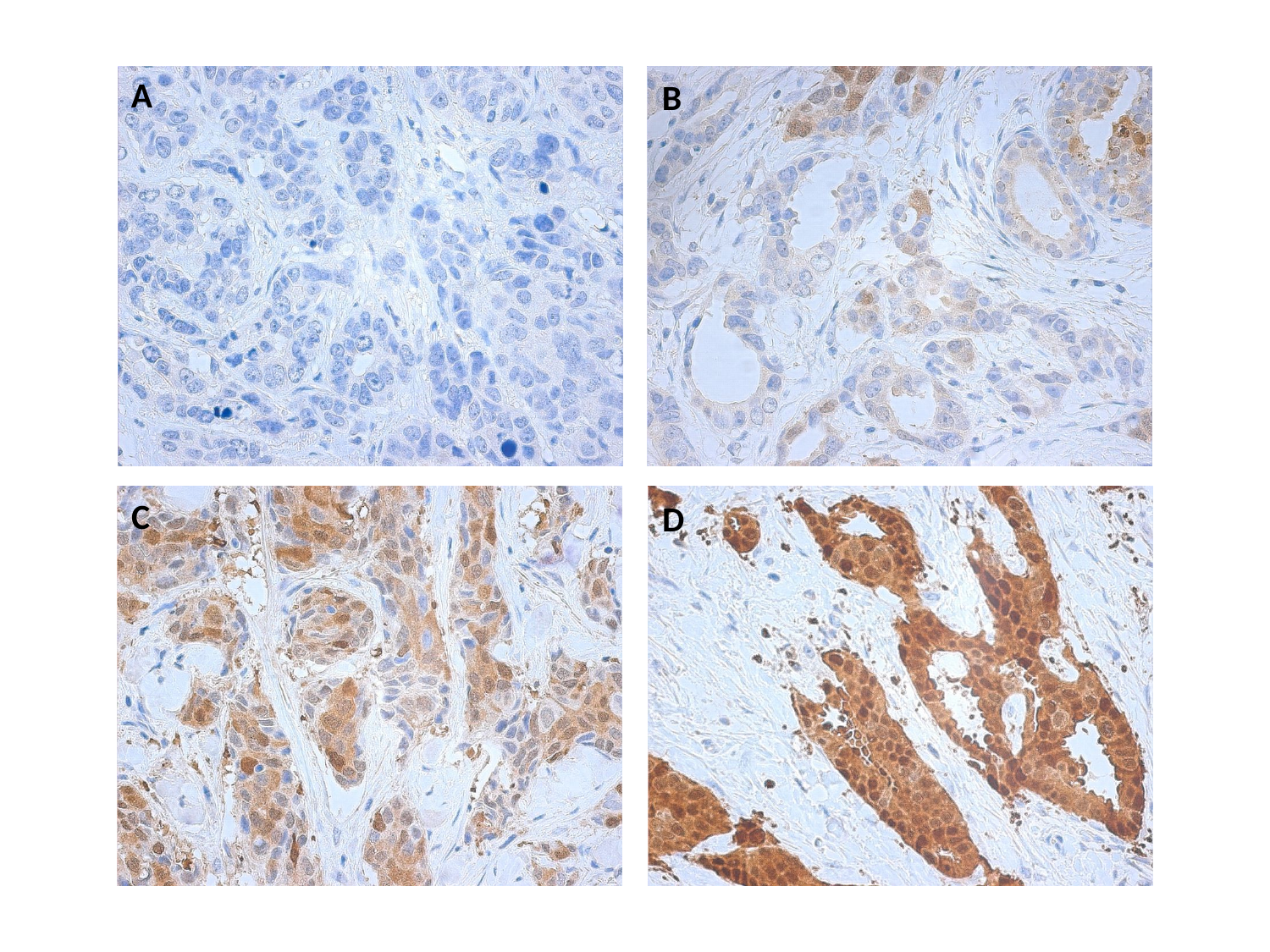

A
B
C
D

Supplement: Supplementary file 2 — Additional file 2: Figure S1: NAT1 immunohistochemical staining of the breast cancer tissues. A, NAT1 expression level was assessed as 0 percent of positively stained tumor cells. B, NAT1 expression level was assessed as 12%. C, NAT1 expression level was assessed as 50%. D, NAT1 expression level was assessed as 75%. x400. (PPTX 6 MB) [file 12885_2014_5180_MOESM2_ESM.pptx]
