# Supplementary material for: Immunohistochemical determination of the miR-1290 target arylamine N-acetyltransferase 1 (NAT1) as a prognostic biomarker in breast cancer
Source: BMC Cancer. 2014 Dec 20;14:990. doi: 10.1186/1471-2407-14-990 (PMC4364092; doi:10.1186/1471-2407-14-990)
Supplement: Supplementary file 3 — Additional file 3: Figure S2: Kaplan-Meier survival analyses of the lymph node negative breast cancer patients. Disease free survival (A) and overall survival (B) of the 161 lymph node positive breast cancer patients stratified according to the presence or absence of NAT1 protein. (PPTX 71 KB) [file 12885_2014_5180_MOESM3_ESM.pptx]

## Slide 1
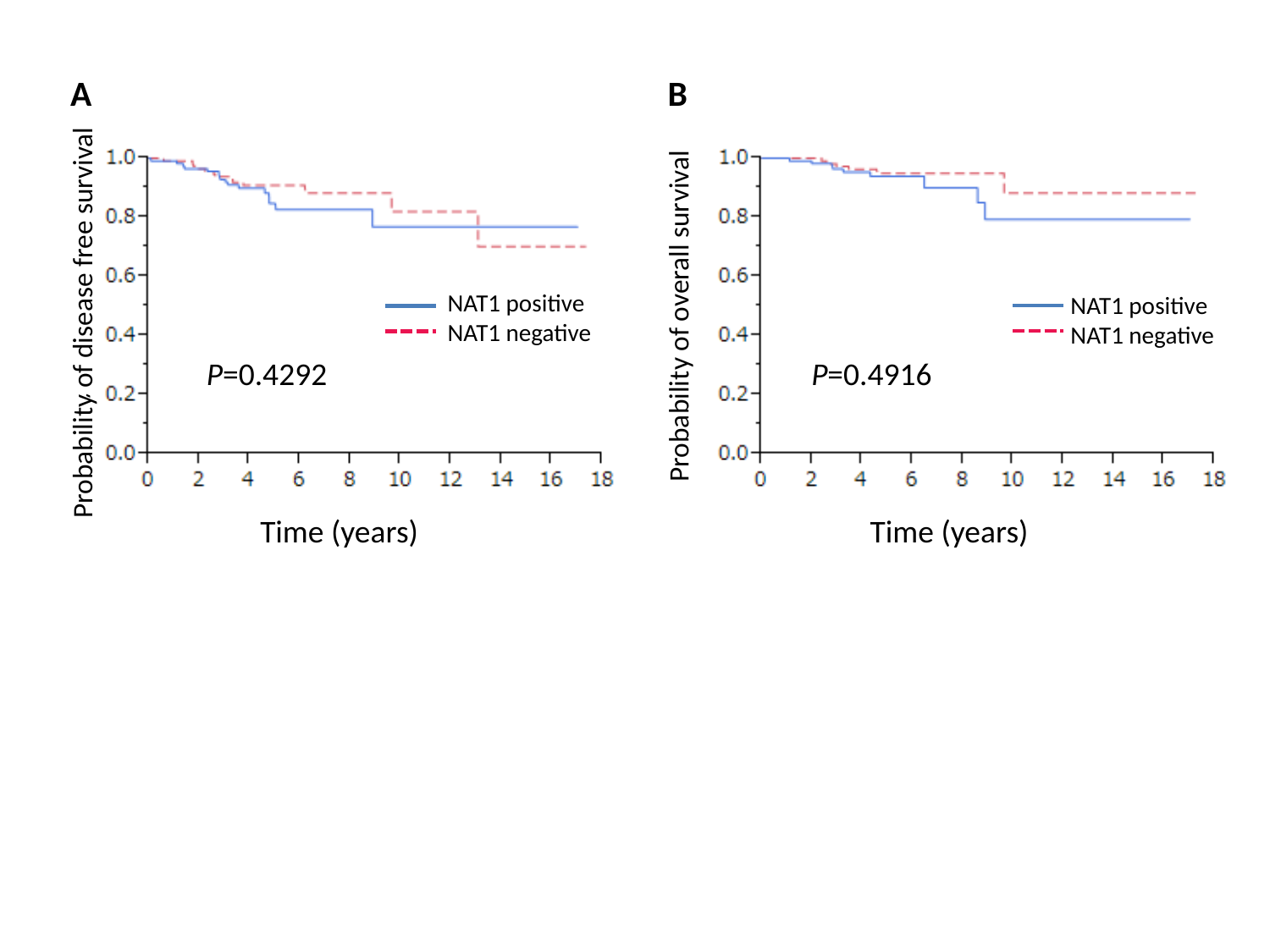

A
B
NAT1 positive
NAT1 negative
NAT1 positive
NAT1 negative
Probability of disease free survival
Probability of disease free survival
Probability of overall survival
Probability of disease free survival
Probability of disease free survival
P=0.4292
P=0.4916
Time (years)
Time (years)
